# Influence of Rolfing Structural Integration on Active Range of Motion: A Retrospective Cohort Study

**DOI:** 10.3390/jcm11195878

**Published:** 2022-10-05

**Authors:** Andreas Brandl, Katja Bartsch, Helen James, Marilyn E. Miller, Robert Schleip

**Affiliations:** 1 Department of Sports Medicine, Faculty for Psychology and Human Movement Science, Institute for Human Movement Science, University of Hamburg, 20148 Hamburg, Germany; 2 Department for Medical Professions, Diploma Hochschule, 37242 Bad Sooden-Allendorf, Germany; 3 Osteopathic Research Institute, Osteopathie Schule Deutschland, 22297 Hamburg, Germany; 4 Department of Sport Science and Sports, Friedrich-Alexander University Erlangen-Nürnberg, 91058 Erlangen, Germany; 5 Department of Physical Therapy, California State University, Fresno, CA 93740, USA; 6 Department of Physical Therapy, University of St. Augustine for Health Sciences at San Diego, San Diego, CA 92069, USA; 7 Conservative and Rehabilitative Orthopedics, Department of Sport and Health Sciences, Technical University of Munich, 80992 Munich, Germany

**Keywords:** active range of motion, structural integration, rolfing, fascia

## Abstract

Background: Recent work has investigated significant force transmission between the components of myofascial chains. Misalignments in the body due to fascial thickening and shortening can therefore lead to complex compensatory patterns. For the treatment of such nonlinear cause–effect pathology, comprehensive neuromusculoskeletal therapy such as the Rolf Method of Structural Integration (SI) could be targeted. Methods: A total of 727 subjects were retrospectively screened from the medical records of an SI practice over a 23-year period. A total of 383 subjects who had completed 10 basic SI sessions met eligibility criteria and were assessed for active range of motion (AROM) of the shoulder and hip before and after SI treatment. Results: Shoulder flexion, external and internal rotation, and hip flexion improved significantly (all *p* < 0.0001) after 10 SI sessions. Left shoulder flexion and external rotation of both shoulders increased more in men than in women (*p* < 0.0001) but were not affected by age. Conclusions: An SI intervention could produce multiple changes in the components of myofascial chains that could help maintain upright posture in humans and reduce inadequate compensatory patterns. SI may also affect differently the outcome of some AROM parameters in women and men.

## 1. Introduction

For many decades, connective tissue, namely the fascia, was seen only as a packaging organ without any active influence on the maintenance of human posture [1]. However, research on this topic over the past 20 years paints a different picture. Schleip et al. [2] discovered active contractile properties of some fascial tissues (e.g., thoracolumbar fascia, gastrocnemius fascia, and fascia lata) that are likely mediated by the sympathetic nervous system. Another mechanism for altering the viscoelastic behavior of such tissues is the ability to change their water content [3]. This offers the possibility of softening or hardening the fascia in the long term and even in the short term (within minutes) depending on various neural or mechanical stimuli. Recent work has led to the knowledge that fascia is an organ that connects rather than separates skeletal muscles [4,5]. Wilke et al. [6] have shown that a force can be transmitted through a sufficiently stiff fascial connection between serially connected skeletal muscles. Therefore, collagenous connective tissue may play an important role in maintaining an upright human posture. We can hypothesize that misalignments in the body due to fascial thickening and shortening can lead to complex compensatory patterns, musculoskeletal disorders, and pain.

Griegel-Morris et al. [7] found a high incidence of postural changes associated with pain in severe cases in 88 healthy adults. Santos et al. [8], in a systematic review and meta-analysis that included 38 studies with a total of 1597 participants studied, attests to the ability of manual therapy interventions to reduce postural asymmetries with moderate certainty. Elkjær et al. [9] demonstrated in a systematic review and meta-analysis that included 73 studies that interventions to reduce contractive postures elicit positive psycho-emotional responses that also contribute to the comprehensive care of affected individuals. Therefore, myofascial force transmission does not depend only on mechanical obstacles in the body tissues such as adhesions between fascial layers, as the ability to apply enough force to change them is questionable [10]. Therefore, the regulation of muscle tone is most likely altered by the modification of neural transmission circuits due to manual therapeutic manipulations, not only at the level of the spine, but also at the level of the motor cortex [11,12]. In addition, there is an intensive psychoneurobiological component in manual therapy that includes expectations, personality traits, learning processes (classical conditioning and observational learning), and mindsets. These mechanisms comprise changes in the endogenous opioid, endocannabinoid, and dopaminergic systems [13].

The Rolf Method of Structural Integration (SI) is a comprehensive “whole body” approach to fascia therapy with elements of sensorimotor education. It focuses on balancing posture by teaching the body to move in a way that is more effective by helping release strain and stiffness [14]. SI was applied in the original form as taught by the founder Dr. Ida Rolf and is still taught at the Dr. Ida Rolf Institute (www.rolf.org, 15 September 2022). SI consists of ten sessions in which the therapist changes the position of each part of the body in relation to the position in space and the gravitational field in order to improve the ergonomics of movements and posture and possibly alleviate the specified complaints [15,16]. Each session lasts approximately 1 h and has its specific course, which usually includes mobilization of all major joints and fascial tissues [16]. The objectives of each session are listed in Table 1. In addition to restoring fascial gliding, the intervention applied by the therapist aims to alter skin receptors and also mechanoreceptors in fascial tissue under the skin (e.g., in the epi/peri/endomysium, fascia profunda, tendons, and joint capsules) [17,18,19]. Jacobson et al. [16] also described an intense psycho-emotional effect of SI. Weinberg et Hunt [20] showed that SI is able to reduce state-trait anxiety. Improvements in the structure of the human body are thought to correlate directly with physiological ameliorations [7,8,18], and fascia also adapts to physical stress, so manual therapy techniques that press on fascia can alter its density, tone, or organization [14,17].

Given the goals of SI and its hypothesized effects on the fascial system, body posture, and psycho-emotional components, SI could result in significant improvement in the active range of motion (AROM) of body joints in patients suffering from myofascial pathologies. However, there is little evidence-based research on structural integration and few high-quality randomized control trials [16,21]. James et al. [22] conducted a retrospective study examining the impact of SI on AROM of the cervical spine and showed that SI can result in a significant decrease in pain and increase in AROM in these structures when used by a physical therapist with advanced SI certification. For this study, the medical records of a local physical therapy practice for cervical AROM and pain data were analyzed. In this work, these records were secondarily analyzed to extract AROM data for the shoulder and hip. We identified six available AROM measurements from the given dataset to evaluate. Although selection was determined by data availability, these tests represent the large joints of the shoulder and hip in addition to the cervical parameters previously examined in the study by James et al. [22] and two multisegment ROM tests. This could reveal evidence of changes involving not only local mechanisms (e.g., fascial tone and lubricity) but also central nervous changes (e.g., psychoneurobiological processes). The purpose of this analysis is to provide comprehensive information on the impact of SI on the AROM of patients in the specific health care setting over a representative period of 23 years of treatment of a private practice.

## 2. Materials and Methods

A secondary analysis of the study data from James et al. [22] was performed. A total of 727 records of subjects were available from the medical records of a local physical therapy practice. Collectively, these subjects were treated by a physical therapist who was also an advanced SI practitioner and a professor emeritus in the Department of Physical Therapy at California State University, Fresno. These prospective subjects were studied between 7 February 1982 and 18 November 2005. All the subjects were given informed consent, and the protocols were approved by the East Orange Department of Veterans Affairs (VA) Medical Center International Review Board (IRB) and the University of Medicine and Dentistry of New Jersey-Newark IRB. All data were coded anonymously in an Excel spreadsheet (Office 2019, Microsoft Corporation, Redmond, WA, USA) so that the investigators could not identify individuals.

### 2.1. Eligibility Criteria

Eligibility criteria were (a) completion of all 10 SI sessions; (b) male or female subjects aged 18 to 60 years; (c) a body mass index (BMI) between 19 and 29 [23]; (d) availability of either shoulder flexion, external rotation, and internal rotation data or hip flexion, side bend (SBN), and finger–floor distance (FFD) data. Subjects without anamnestically reported medical conditions were excluded.

### 2.2. Outcomes

Subjects were assessed for complaints related to back, neck, and other pain syndromes or combinations of such syndromes. AROM measurements were taken during the initial (before the start of SI treatment sessions) and final (after 10 SI sessions) assessments. The entire SI therapy was performed in an average time of 6 months in each patient. Clinical data collected included age, sex, dates of initial and final assessments, complaints before, during, and after SI, diagnosis, height, weight, and AROM. Data were collected during the initial assessment by a physical therapist with more than 20 years of experience in AROM measurement and provided the basis for determining subjects’ functional limitations, interventions, and outcomes.

All AROM measurements except for SBN and FFD were performed with the patient supine using a 12-inch 360° goniometer labeled in 1° increments, with two adjustable overlapping arms.

#### 2.2.1. AROM of the Shoulder

Shoulder flexion ROM was measured by instructing the patient to raise their arm straight above their head as far as possible. The stationary arm was positioned parallel to the midline of the rib cage, while the movable arm was aligned with the shaft of the humerus and the lateral epicondyle [24]. Intra-rater reliability according to Fleiss [25] was reported to be excellent (ICC = 0.95), and the minimal detectable change (MDC) was 4.8° [26].

External rotation of the shoulder was measured by passively placing the patient’s arm in 90° abduction with the elbow flexed at 90° and instructing the patient to rotate the arm as far back as possible with the palm pointing toward the ceiling. Goniometric standard positioning was used by placing the stationary arm perpendicular to the floor and aligning the moving arm with the shaft of the ulna and the styloid process [24]. Intra-rater reliability was reported to be excellent (ICC = 0.98), and the MDC is 8.1° [26].

Internal rotation of the shoulder was measured by passively placing the patient’s arm in 90° abduction with the elbow flexed at 90° and instructing the patient to rotate his or her arm forward as far as possible so that the palm was pointing toward the floor. Goniometer positioning for measurement was also performed in a standardized manner [24]. Intra-rater reliability was reported to be excellent (ICC = 0.94), and the MDC is 6.8° [26].

#### 2.2.2. AROM of the Hip

**Figure 1:**
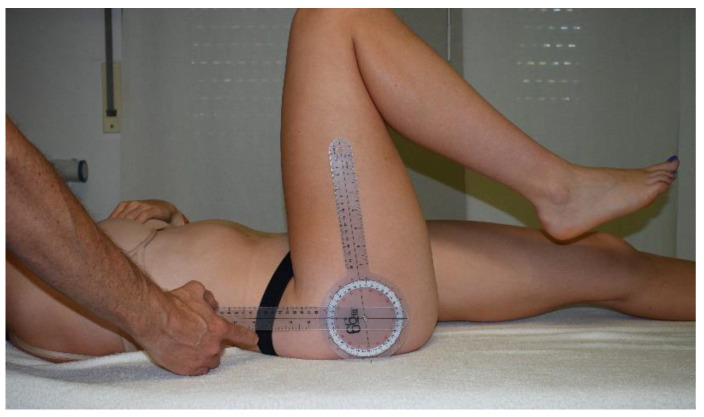
Hip flexion measurement.

Hip flexion ROM was measured by instructing the subject to keep the contralateral distal thigh stable toward the treatment bench and to avoid pelvic motion during the test. The participant was then asked to flex the hip with the knee in flexion until they felt a firm end sensation or pain restriction that prevented further movement. The examiner then aligned the axis of the goniometer with the greater trochanter and the arms with the lateral condyle of the thigh and the midaxillary line. When the trunk and thigh were parallel, hip flexion AROM was defined as 0° (Figure 1). Subjects held their hands across the chest during the test. Intra-rater reliability in measuring hip joint range of motion was reported to be good (ICC = 0.75), and the MDC is 13° [27].

#### 2.2.3. Side Bend

To evaluate trunk movement during lateral bending, the subject stood upright on the floor, heels together, knees straight, and arms in a neutral position. The subject was asked to bend to the side with arms hanging as far as possible while keeping the fingers straight. Then, the distance between the tip of the right or left third finger and the floor was measured with a ruler. The reliability coefficients for these measurements were reported to be large (r = 0.91), and the MDC is 3.6 cm [28].

#### 2.2.4. Finger to Floor Distance

The distance between the fingers and the floor was also measured while the subject stood upright on the floor, heels together, knees extended and arms in a neutral position. The subject was asked to bend forward as far as possible while keeping the knees and fingers straight. Then, the distance between the tip of the right 3rd finger and the floor was measured with a ruler. The reliability coefficients for these measurements were reported to be high (r = 0.98) and the MDC is 3.7 cm [28].

### 2.3. Statistical Analyses

The standard deviation (SD), mean, and 95% confidence interval (95% CI) were determined for all parameters. Outliers above 1.5 times the interquartile range of the third quartile or below this factor of the first quartile were excluded from the analysis. Variables that did not meet the assumptions of normal distribution according to the Kolmogorov–Smirnov test (*p* < 0.05) or violated homogeneity of error variances between groups according to Levene’s test (*p* < 0.05) were Box–Cox transformed. A four-way ANOVA with the factors Structural Integration (SI) treatment (before and after), sex (female and male), age group (younger and older or equal to the median age), and illness (back pain, neck pain, shoulder pain, combined, and other) was used to test the hypotheses. Partial eta squared was calculated as effect size and interpreted according to Cohen [29] as small (0.01–0.05), medium (0.06–0.13), and large (>0.13). Significant interaction effects were assessed using the Tukey’s HSD post hoc test analysis. The significance level was set at *p* = 0.05.

Libreoffice Calc version 6.4.7.2 (Mozilla Public License v2.0) was used for the descriptive statistics. The inferential statistics were carried out with R software, version 3.4.1 (R Foundation for Statistical Computing, Vienna, Austria).

## 3. Results

**Figure 2:**
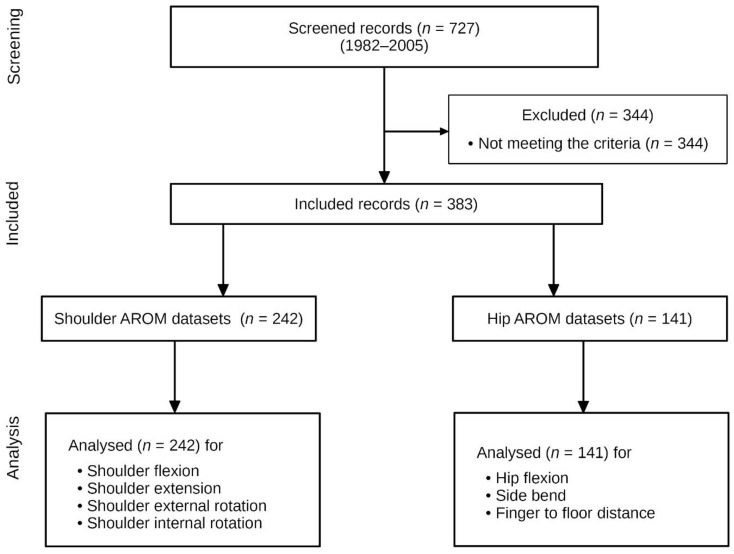
Flow chart of the study.

The anthropometric data and baseline characteristics are shown in Table 2. Of 727 subjects treated between 7 February 1982 and 18 November 2005, 383 met the eligibility criteria and were analyzed (Figure 2). Thirty-five subjects did not meet the age requirements, 84 were not within the acceptable BMI range, and not all data were available from 230 subjects. The median age was 39.8 years.

For all outcomes, there was no four-way interaction between SI treatment, gender, age group, and disease. There was no three-way interaction between SI treatment, gender, and age group; SI treatment, gender, and disease; or SI treatment, age group, and disease (all *p* > 0.05).

**Figure 3:**
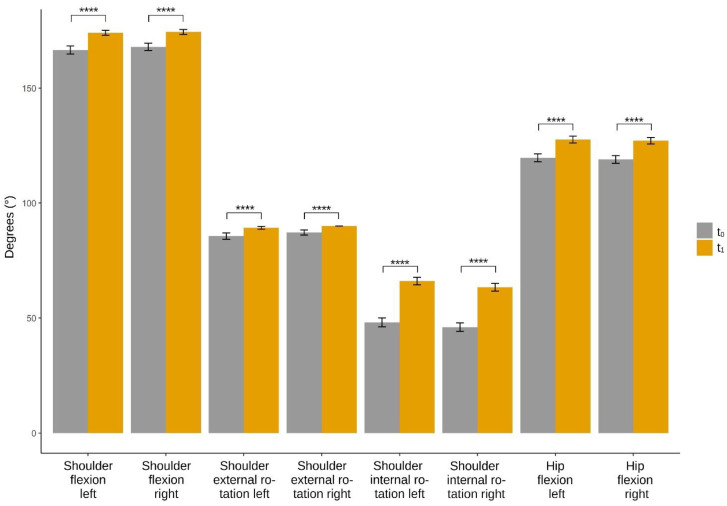
Changes before and after Rolfing Structural Integration treatment. t_0_, baseline measurement; t_1_, measurement after treatment. The error bars show the 95% confidence interval. Significant at the level **** <0.0001.

The four-way ANOVAs revealed a significant two-way interaction between SI treatment and gender for left (F(1, 418) = 8.97, *p* = 0.003, partial η² = 0.020) but not for right (F(1, 416) = 2.96, *p* < 0.086, partial η² = 0.007) shoulder flexion. There were no significant interactions between treatment and age group or treatment and disease (all *p* > 0.05). There was also a significant interaction between SI treatment and sex for left (F(1, 386) = 10.63, *p* = 0.001, partial η² = 0.027) but not for right (F(1, 440) = 1.05, *p* < 0.305, partial η² = 0.002) shoulder external rotation, but there were no significant interactions between treatment and age group or treatment and disease (all *p* > 0.05). For all other outcomes, there were no significant two-way interactions (all *p* > 0.05). A post hoc Tukey’s HSD test was performed for the significant interactions. The results of the post hoc analysis and the simple main effects are shown in Table 3 and Figure 3.

## 4. Discussion

This secondary analysis of the study data from James et al. [22] aimed to provide comprehensive information on the impact of SI on patients’ shoulder and hip AROM. This retrospective study, along with the work of James et al. [22], was the first to examine SI in 727 subjects in the specific health care setting of a private practice over a representative 23-year period.

### 4.1. AROM of the Shoulder

The main results showed that all measured AROM parameters of the shoulder were significantly increased after SI treatment. Flexion and external rotation showed a significant interaction between gender and treatment. However, while the mean flexion difference was about 3° above the MDC, the external rotation was about 3.5° below. It is noteworthy at this point that AROM increased much more for men than for women for the left shoulder (e.g., +108% vs. +40% for left shoulder flexion). It is well-known that myofascial stiffness is higher in men than in women [30]. A recent study by Bohlen et al. [31] found increased efficacy of more force-intensive manual therapy techniques on muscle tone at rest in men than in women. There is also evidence of higher lumbar myofascial stiffness on the dominant side of the body [32,33]. Data on hand dominance were not available in this study. However, it can be assumed that most participants were right-handed, as only 10% of people have left-hand dominance [34]. Considering the stiffness of the thoracolumbar fascia (TLF) and its ability to transfer force from the right to the left side of the body [35,36,37], SI-induced reduction in TLF stiffness could lead to improvement in left shoulder flexion in addition to local mechanisms. However, SBN and FFD, for which we found no change, would remain unchanged with this mechanism. This is consistent with Brandl et al. [38], who also found no changes in FFD but other postural changes after manual treatment of the thoracolumbar fascia.

The movement in the shoulder that improved the most was internal rotation. An amelioration of 17° to 18° was achieved, corresponding to an increase of 37–38% when compared before and after SI treatment. The result exceeded the MDC by over 9°. James et al. [22], referring to the same data, reported the highest increase in motion in lateral neck flexion (32%). One muscle that is highly involved in both movements is the trapezius muscle [39]. Although both functions are influenced to varying degrees by different parts of this muscle, it may be possible that SI, in addition to various other neuromuscular improvements, altered the functionality of the trapezius, which could lead to an enhancement of both AROM parameters. Future studies are needed to investigate such hypothesized relationships.

### 4.2. AROM of the Hip

For hip (and trunk) mobility, the results showed a significant improvement in flexion AROM of both hip joints (+7%). No interaction was found between gender and treatment. This was surprising, as there were such interactions in some shoulder parameters, indicating different efficacy of SI treatment in men and women. Considering the gender differences in response to manual therapy techniques and TLF stiffness, one might expect the results to be similar in the hip and shoulder [30,31]. It is worth noting that the mean hip flexion difference was 2.7° under the MDC. Therefore, future research focusing on the gender-specific efficacy of SI and clinically relevant changes is needed to uncover further associations of these mechanisms.

### 4.3. General Effects

It is well-known that force can be transmitted via myofascial continuities [6,40]. In a systematic review of cadaveric studies, Krause et al. [40] investigated that the posterior myofascial chain in particular is capable of transmitting significant forces from the plantar aponeurosis via the gastrocnemius, the hamstring muscles to the TLF and the erector spinae muscle. Wilke et al. [6] also confirmed these findings in vivo. It is possible that SI treatment influenced these myofascial force transmission systems. Therefore, the restoration of inadequate gliding properties of muscles, tendons, nerves, and soft tissues could be a specific SI effect [41]. It is likely that sciatic nerve stiffness plays a critical role in hip motion. Mechanical forces acting on peripheral nerves could alter structures far beyond the moving joint [42]. Therefore, it could be possible that SI treatments acting on the sciatic nerve could improve hip flexion.

Manual therapy techniques such as Myofascial Release or Osteopathic Manipulative Treatment have an immediate impact on trunk and pelvic shape parameters [8,18]. It is likely that these changes are due to immediate neuromotor muscle changes, so one may ask whether SI has a similar effect that lasts over a longer period of time, as shown by the results of this study [43]. Here, SI treatment may have altered mechanoreceptors in fascial tissue (e.g., in the epi/peri/endomysium, fascia profunda, tendons, and joint capsules). These may have triggered changes in muscle tone, hydration, and neurological effects, which was likely achieved by the interventions in this study [17,19].

There were no significant effects of the interventions on SBN and FFD, but there was a trend for a slight increase in right SBN (+2%, *p* = 0.05) and a decrease in FFD (−25%, *p* = 0.08). Even though there was only a statistical trend, it was surprising, as this parameter was also expected to increase by the mechanisms mentioned above. Chen et al. [44] found no changes in FFD after seven weeks of flexibility training in participants with low flexibility, but their results showed that erector spinae and hamstring activation, pelvic tilt, and lumbosacral angle were significantly altered after training. This is consistent with the results of this study, in which most of the studied AROM parameters except FFD increased. 

SI has an intense psycho-emotional component in addition to the mechanical influences on soft tissue [16]. Therefore, this observational study can only provide results and hypothetically discuss possible SI-specific effects to provide clues for future experimental studies. It remains unclear whether the results of this retrospective study are based only on soft tissue manipulation or movement education and the psychoneurobiological component involved in daily practice and interpersonal contact between therapist and patient [9].

### 4.4. Age-Related Treatment Effects

It is known that the fasciae lose flexibility and become thicker with age [45]. Hence the hypothesis that SI, as a treatment method that acts on this tissue in particular, has a different effect in older people than in younger people. However, in contrast to James et al. [22], this study did not show age-related treatment effects. No interaction between age and treatment was found in the four-way ANOVA. Groups were divided into younger and older subjects based on the mean age of 40 years. In the study by James et al., the mean age was much higher, 52 years. It is possible that the discrimination age of 40 years was not sufficient to create two groups with appreciable enough age-related differences [46] to detect treatment interactions between older and younger subjects. Further studies need to take this into account and therefore choose a different age of discrimination or even form more than two age groups.

### 4.5. Limitations

The secondary analysis study presented here had the strengths mentioned above, particularly enhanced external validity due to data acquisition of patients in a running practice over a representative period of 23 years, but also the limitations of a retrospective uncontrolled study.

Randomization was not possible, as is the case in most observational studies. Therefore, a selection bias might be present because people with certain characteristics might be more attracted to a specific therapy method like SI than others. However, the study included a large number of subjects and was conducted over a representative study period of 23 years. Therefore, the results could at least be generalizable to a population using SI health care.

Pain data were not available in the patients’ medical records evaluated. James et al. [22] included these data and provided results for this in a previous study. There was also no control group to which we could compare the SI-treated subjects, no follow-up data, and participants were not controlled for receiving other treatments during an average of six months of SI therapy. Therefore, the study can only provide limited information on the clinical relevance of the results. Future studies on SI should consider a control-group design and include follow-up data in their designs. It is furthermore recommended for future work to collect patient-centered information on disability improvements and functional enhancements (e.g., Oswestry Disability Questionnaire, Tinnetti Performance-Oriented Assessment of Mobility, Berg Balance Scale, Fullerton Advanced Balance Scale, Dynamic Gait Index, Balance Efficacy Scale, Pain Disability Index).

The aim of this work was to extend the cervical spine AROM parameters investigated in a previous study for the shoulder and hip. According to Abbott et al. [47], such observations represent an appropriate design for this type of study to quantify AROM changes due to SI treatment. However, it is difficult to infer causality from such study designs. This must be seen considering the lack of SI studies to date (only 13 results are listed on PubMed with the search string: “(“structural integration”) AND (rolf OR rolfing)”. While Structural Integration is becoming more organized as a profession, the amount of evidence-based research on Structural Integration remains limited [16,21]. A current review of studies on body-centered interventions for psychopathological conditions points to the scarcity of research and small sample sizes related to Structural Integration [48]. Although the level of evidence from this observational study is below that of a randomized control trial, the results are particularly useful given the paucity of studies that have examined the efficacy of SI therapy and may also provide valuable baseline information for the development of subsequent comprehensive, high-quality studies.

## 5. Conclusions

This work suggests that 10 SI sessions, when delivered by a physical therapist with advanced SI certification, could increase shoulder and hip mobility. It is likely that SI interventions produce multiple changes in the components of myofascial chains that could help maintain upright posture in humans and reduce inadequate compensatory patterns. SI may also affect the outcome of the AROM parameters of left shoulder flexion and external rotation of both shoulders differently in women and men. Further investigation is needed to determine if similar or more advanced results are also obtained in an experimental setting with a randomized control trial design.

## Figures and Tables

**Table 1:** Description of ten Structural Integration (SI) interventions.

Session	Intervention
1	The focus of this session is breathing. Myofascial release is applied to the muscles of the trunk, ribs, shoulder, neck, and head. They also mobilize the hip and legs to align the pelvis horizontally. The assessment and treatment of specific problems is addressed throughout every session.
2	The focus of this session is posture. They balance the feet, lower legs, and knees to correct the spinal curvatures.
3	The focus of this session is spinal elongation. They work on the lateral aspect of the body to create elongation of the spine.
4	The focus of this session is stability of the legs. They work on the plantar arches and the medial aspect of the lower extremity. The spine is mobilized and continues to be elongated.
5	The focus of this session is to balance the trunk with the legs. They work on the abdominal, pelvic, and iliopsoas muscles. The outer abdominal wall is elongated and mobilized to create continuity with the inner tissues.
6	The focus of this session is to improve trunk mobility with stabilization of the pelvis and lower extremities. They work on the posterior pelvis, back, neck, and head.
7	The focus of this session is to balance the rhythm of cranial movements. Work is done in the upper back, shoulders, neck, cranium, and facial structures.
8	The focus of this session is lower body integration. Work is done to the pelvic girdle and extremities. Specific problems become a priority in the treatment.
9	The focus of this session is upper body integration. Work is done to the pelvic girdle and extremities. Specific problems become a priority in the treatment.
10	The focus of this session is to correct the level of the structures bilaterally during static and dynamic activities. Specific problems become a priority in the treatment. Specific problems are assessed and treated throughout the 10 sessions.

A detailed description of the modalities and goals of Rolfing Structural Integration can be found in Jacobsen et al. [16].

**Table 2:** Baseline characteristics.

Baseline Characteristics	Participants (*n* = 383) Mean ± SD
Gender (men/woman)	154/228
Disease (A/B/C/D/E)	101/114/42/27/99
Age (years)	39.0 ± 11.1
Age group (younger/older)	189/194
Height (m)	1.70 ± 0.1
Weight (kg)	71.3 ± 16.1
BMI (kg/m^2^)	25.2 ± 5.7

SD, standard deviation; *n*, number; Disease: A, other; B, back pain; C, neck pain; D, shoulder pain; E, combined A to D.

**Table 3:** Descriptive statistics and ANOVA.

	All Subjects (*n* = 383)										
	Shoulder (*n* = 242)						Hip (*n* = 141)		Functional Tests (*n* = 141)		
	Flexion L	Flexion R	External Rotation L	External Rotation R	Internal Rotation L	Internal Rotation R	Flexion L	Flexion R	Side Bend L	Side Bend R	Finger to Floor
∆Mean	7.81°	7.16°	4.26°	4.52°	18.00°	17.33°	8.30°	8.26°	0.42 cm	0.68 cm	−2.21 cm
SD	12.87	10.83	9.06	10.61	13.13	10.87	8.84	8.70	1.61	1.77	6.73
95%-CI	5.17–10.6	4.62–9.70	2.61–5.92	2.22–6.22	15.5–20.5	14.9–19.8	6.12–10.8	6.14–10.9	−0.33–1.17	−0.07–1.42	−4.51–0.09
% t_1_–t_0_	+5%	+4%	+5%	+5%	+37%	+38%	+7%	+7%	+1%	+2%	−25%
ANOVA ^1^											
DFn, DFd	1418	1416	1386	1386	1440	1440	1238	1242	1242	1244	1230
F	70.80	57.26	70.80	32.81	207.7	189.3	56.41	57.48	1.56	3.75	3.02
part. η²	0.15	0.12	0.08	0.08	0.32	0.30	0.19	0.19	0.006	0.015	0.013
*p*	<0.0001	<0.0001	<0.0001	<0.0001	<0.0001	<0.0001	<0.0001	<0.0001	0.213	0.054	0.084
	Female (*n* = 228)										
	Shoulder (*n* = 147)						Hip (*n* = 81)		Functional tests (*n* = 81)		
	Flexion L	Flexion R	External rotation L	External rotation R	Internal rotation L	Internal rotation R	Flexion L	Flexion R	Side bend L	Side bend R	Finger to floor
∆Mean	5.48°	6.19°	2.46°	3.45°	16.97°	16.64°	8.70°	8.21°	0.43 cm	0.67 cm	−1.93 cm
SD	14.34	11.29	7.34	10.59	13.69	10.46	8.76	8.11	1.60	1.79	6.82
95%-CI	1.04–9.91	1.92–10.5	−0.34–5.25	0.62–6.32	12.8–21.1	12.5–20.8	4.94–12.5	4.54–11.9	−0.84–1.69	−0.61–1.95	−5.91–2.06
*p* (adjusted) ^2^	0.008		0.158	0.112							
	Male (*n* = 154)										
	Shoulder (*n* = 95)						Hip (*n* = 59)		Functional tests (*n* = 59)		
	Flexion L	Flexion R	External rotation L	External rotation R	Internal rotation L	Internal rotation R	Flexion L	Flexion R	Side bend L	Side bend R	Finger to floor
∆Mean	11.42°	8.65°	7.06°	6.16°	19.58°	18.40°	7.75°	8.33°	0.42 cm	0.69 cm	−2.59 cm
SD	9.16	9.94	10.66	11.31	12.13	11.46	8.99	9.51	1.64	1.77	6.65
95%-CI	5.90–16.94	3.33–14.0	3.59–10.5	2.61–9.70	14.4–24.7	13.3–23.6	3.38–12.1	4.07–12.6	−1.05–1.89	−0.82–2.19	−7.22–2.04
% m–f	+108%	+40%	+187%	+79%	+15%	+11%	−11%	+1%	−2%	+3%	+34%
*p* (adjusted) ^2^	<0.0001		<0.0001	<0.0001							

∆Mean, mean difference before and after Rolfing Structural Integration treatment; SD, standard deviation; *n*, number; 95%-CI, 95% confidence interval; % t_1_–t_0_, percentage difference between baseline and after treatment; % m–f, percentage difference between men and women; DFn, degree of freedom for the numerator; DFd, degree of freedom for the denominator; *p*, *p*-value; part. η², partial η²; L, left; R, right. ^1^ Simple main effect of Rolfing Structural Integration treatment. ^2^ Adjusted *p* from Tukey’s HSD test for significant treatment/gender interactions.

## Data Availability

Data can be made available by the author upon request.
